# Papillary atrophy of the tongue

**DOI:** 10.1002/ccr3.1808

**Published:** 2018-09-12

**Authors:** Nozomi Niimi, Nobuaki Mori

**Affiliations:** ^1^ Department of General Internal Medicine National Hospital Organization Tokyo Medical Center Tokyo Japan

**Keywords:** anemia, gastrectomy, papillary atrophy, vitamin B12 deficiency

## Abstract

Do not forget to give cobalamin supplementation after gastrectomy. Patients at risk of vitamin B12 deficiency should have their vitamin B12 level monitored routinely.

## CLINICAL IMAGE

**Figure 1:**
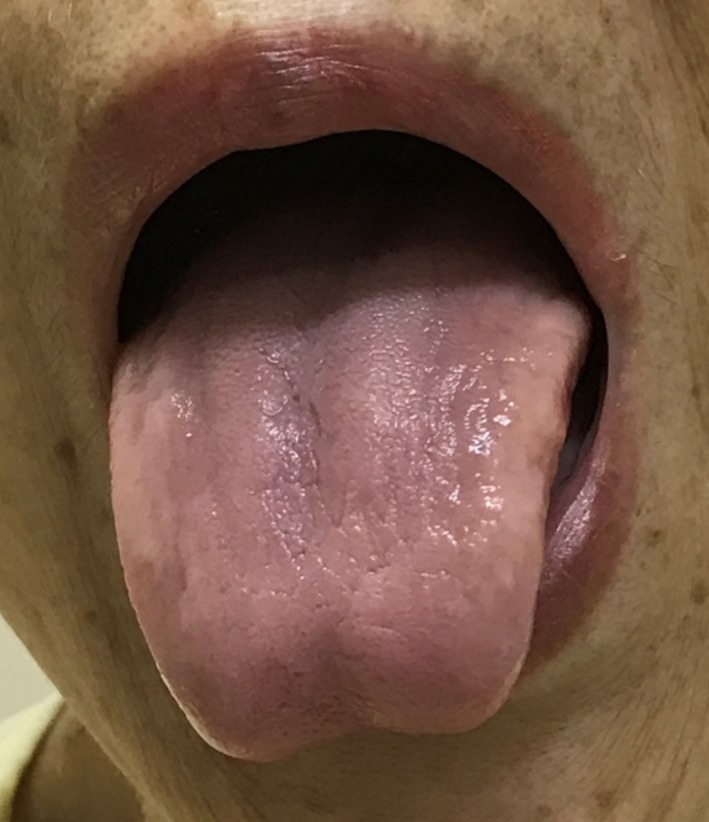
Atrophy of the tongue papillae with vitamin B12 deficiency.

**Figure 2:**
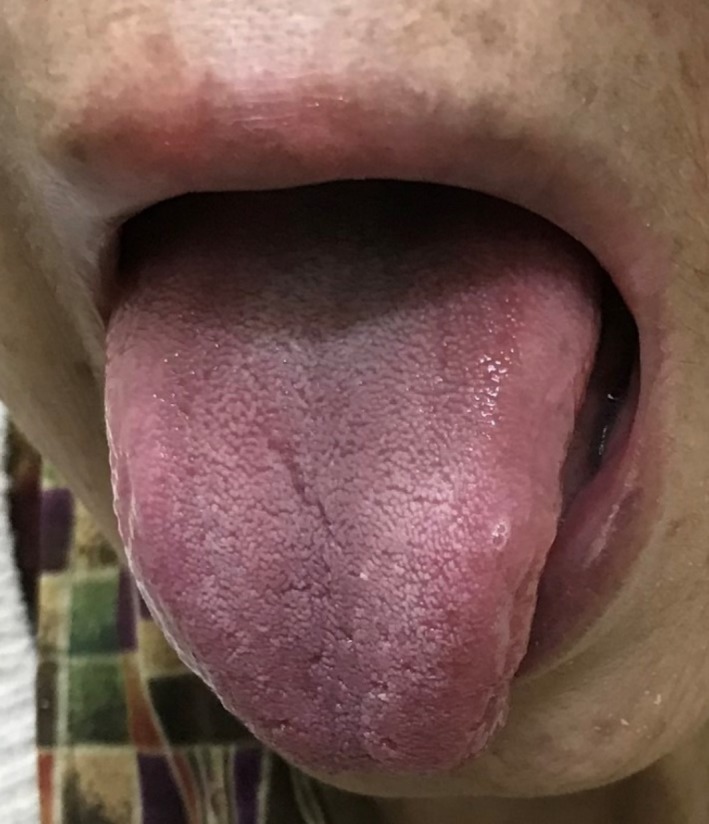
Normal appearance of the tongue after vitamin B12 asupplementation.

A 73‐year‐old woman presented to the outpatient department with anemia which was identified during preoperative examination of cataract. The patient had undergone total gastrectomy 10 years ago without cobalamin supplementation. On examination, she had atrophy of the tongue papillae (Figure 1) and moderate dementia. Laboratory tests showed macrocytic anemia (hemoglobin 8.2 g/dL, mean corpuscular volume 123 fL) and her serum vitamin B12 level was 110 pg/mL (range: 233‐914 pg/mL). Vitamin B12 deficiency was diagnosed. She started cobalamin supplementation and her anemia, tongue papillae, and cognitive function improved 2 months later (Figure 2).

After total gastrectomy, patients are at high risk of vitamin B12 deficiency due to decreased absorption; therefore, cobalamin supplementation is indicated. Papillary atrophy is an important finding characteristics of vitamin B 12 deficiency. Tongues with vitamin B 12 deficiency have a smooth, glossy appearance with a red or pink background.^1^ The etiology of papillary atrophy includes both local diseases, such as oral candidiasis and chemical irritantation, and systemic diseases such as nutrition deficiencies, amyloidosis, and celiac disease.^1^ Physicians should examine the patient's history and other symptoms for differential diagnosis. Oral lesions have been reported in 25% of all patients with megaloblastic anemia, and they may precede other symptoms.^2^ Examing patients’ mouth will reveal much disease‐related information.

## CONSENT FOR PUBLICATION

Written informed consent was obtained for the publication of this clinical picture.

## AUTHOR CONTRIBUTION

NN: cared for the patient and wrote the report. NM: reviewed the paper and provided recommendation. All the authors approved the final version submitted to the journal.

## CONFLICT OF INTEREST

None declared.
